# Contrasting effects of ploidy level on seed production in a diploid–tetraploid system

**DOI:** 10.1093/aobpla/plw077

**Published:** 2016-12-24

**Authors:** Zuzana Münzbergová, Jiří Skuhrovec

**Affiliations:** 1 Department of Botany Faculty of Science, Charles University, Prague, Czech Republic; 2 Institute of Botany, Academy of Sciences of the Czech Republic, Průhonice, Czech Republic; 3 Group Function of Invertebrate and Plant Biodiversity in Agro-ecosystems, Crop Research Institute, Prague, Czech Republic

**Keywords:** AMOS, Asteraceae, Carduoideae, pollinators, polyploid, pre-dispersal seed predation, seed set, structural equation modelling

## Abstract

Previous studies demonstrated the effects of polyploidy on various aspects of plant life. It is, however, difficult to determine which plant characteristics are responsible for fitness differences between cytotypes. We assessed the relationship between polyploidy and seed production. To separate the effects of flowering phenology, flower head size and herbivores from other possible causes, we collected data on these characteristics in single flower heads of diploid and tetraploid *Centaurea phrygia* in an experimental garden. We used structural equation modelling to identify the main pathways determining seed production. The results showed that the relationship between polyploidy and seed production is mediated by most of the studied factors. The different factors acted in opposing directions. Wider flower heads displayed higher above the ground suggested higher seed production in diploids. In contrast, earlier flowering and a lower abundance of herbivores suggested higher seed production in tetraploids. However, because phenology was the strongest driver of seed production in this system, the sum of all the pathways suggested greater seed production in tetraploids than in diploids. The pathway linking ploidy level directly to seed production, representing unstudied factors, was not significant. This suggests that the factors studied likely are drivers of the between-cytotype differences. Overall, this study demonstrated that tetraploids possess overall higher fitness estimated as seed production. Regardless of the patterns observed here, strong between year fluctuations in the composition and diversity of insect communities have been observed. The direction of the selection may thus vary between years. Consequently, understanding the structure of the interactions is more important for understanding the system than the overall effects of cytotype on a fitness trait in a specific year. Such knowledge can be used to model the evolution of species traits and plant-herbivore and plant-pollinator interactions in diploid–polyploid systems.

## Teaser

The relationship between polyploidy in diploid and tetraploid Centaurea phrygia and seed production is mediated by a wide range of factors with opposing directions. Wider flower heads displayed higher above the ground suggested higher seed production in diploids while earlier flowering and a lower abundance of herbivores suggested higher seed production in tetraploids. The importance of different relationships varies between years. Knowledge of the patterns of interactions is thus more important than the specific values of the links and can be used to model the evolution of species traits and plant-herbivore and plant-pollinator interactions in diploid-polyploid systems.

## Introduction

Determining the consequences of polyploidy is a key prerequisite for assessing the importance of this mechanism of speciation. To do this, an increasing number of studies address the consequences of polyploidy in various aspects of plant life ranging from plant morphology and physiology to individual plant performance, population dynamics and habitat requirements (recently reviewed in Ramsey and Ramsey 2014), although most studies focused on established polyploids and thus struggle to decouple effects of polyploidization from subsequent evolutionary change. Recently, it has been recognized that polyploidy also affect the interactions of plants with other trophic levels such as herbivores, pollinators and mycorrhizal fungi (recently reviewed in Segraves and Anneberg 2016). Among herbivores, the species feeding on reproductive plant structures are the most commonly studied (e.g. Thompson *et al. *1997; Münzbergová 2006a; Arvanitis *et al. *2010), likely due to their strong direct effects on fitness.

Based on the previous studies dealing with the effects of polyploidy on plant-herbivore and plant-pollinator interactions, we already know that polyploidy may have substantial effects. The mechanisms behind these effects are, however, still only poorly understood. In general, plant-herbivore and plant-pollinator interactions may be affected by a wide range of factors ranging from individual plant characteristics such as plant height, floral rewards, flower size, flower duration, flower position, phenology and the chemical composition of plant tissues (e.g. Loranger *et al. *2012; Münzbergová and Skuhrovec 2013; Gross and Schiestl 2015; Schlinkert *et al. *2015; Smith *et al. *2015) to characteristics of plant surroundings, such as vegetation height or conspecific and heterospecific density and diversity (e.g. Weber and Kolb 2013; Burt *et al. *2014; Červenková and Münzbergová 2014; Hulber *et al. *2015; Kim and Underwood 2015) and to population characteristics, such as population size or density (e.g. Münzbergová 2006a; Sober *et al. *2009).

Individual-level characteristics, such as flowering phenology and flower size and duration, are plant traits commonly reported as being affected by polyploidy and may thus be responsible for the differential associations of different cytotypes with pollinators and herbivores that feed on reproductive structures (e.g. Jersáková *et al. *2010; Konig *et al. *2015; Münzbergová *et al. *2015). Among these traits, flowering phenology is expected to be a key mechanism driving the differential interactions between different plant cytotypes and the herbivores and pollinators given the short life-span and the specific timing of reproduction in many invertebrates (Schoonhoven *et al. *1998; Ehrlén and Münzbergová 2009) that are linked to species-specific requirements for effective temperatures (Trudgill *et al. *2005; Martínková and Honěk 2008). Each herbivore and pollinator is thus likely to occur only within a limited window of time within the vegetation season. Consequently, differences in flowering phenologies may lead to different plant–herbivore and plant–pollinator interactions among different cytotypes and potentially be under strong selection as a consequence of these interactions (e.g. Nuismer and Cunningham 2005; Perez-Barrales *et al. *2013; Jordan *et al. *2015; Sletvold *et al. *2015). Only a study by Nuismer and Cunningham (2005) has, however, explicitly assessed the intensity of selection on traits in different cytotypes.

In addition to the importance of flowering phenology for differential plant–herbivore and plant–pollinator interactions among cytotypes, flowering phenology is also an important pre-reproductive barrier among different cytotypes (e.g. Petit *et al. *1997; Castro *et al. *2011), leading to additional selection pressures on flowering phenology in many polyploid systems (e.g. Van Dijk and Bijlsma 1994; Petit *et al. *1999; Nuismer and Cunningham 2005), even though other reproductive barriers may sometimes be more important (Husband and Sabara 2004).

While flowering phenology seems to be a likely explanation for the differences in the plant–herbivore and plant–pollinator interactions between different cytotypes with likely consequences for plant fitness, most existing data do not allow the testing of this assumption. This is because the effects of phenology and ploidy level are closely related to one another, making their proper separation difficult. A close relationship between ploidy level and phenology was previously shown for diploid and tetraploid *Chamerion angustifolium* (Husband and Schemske 2000), tetraploid and octoploid *Gymnadenia conopsea* (Jersáková *et al. *2010), diploid and hexaploid *Aster amellus* (Castro *et al. *2011) and diploid and tetraploid *Centaurea phrygia* (Münzbergová *et al. *2015).

In our previous study, we demonstrated strong differences in the composition of herbivore communities and in seed damage in the flower heads of diploid and tetraploid cytotypes of *Centaurea phrygia* (Münzbergová *et al. *2015). The two cytotypes were shown to differ in a large number of traits including flower head size, the content of secondary metabolites and flowering phenology. However, all these traits were measured only at the population level and were not directly related to the sampled flower heads, thus making it impossible to identify the key drivers of plant–herbivore interactions. In addition, the previous study looked only at seed herbivores and seed damage neglecting the fitness of the different cytotypes.

In this study, we aimed to assess the independent effects of flowering phenology and flower head traits (flower head height above the ground and flower head width) on the composition of herbivore communities in the flower heads and on seed damage and seed production in the two cytotypes by studying detailed phenology at the level of single flower heads. In this way, we were able to obtain data from simultaneously flowering flower heads of the two cytotypes and were thus able to obtain sufficient power to study the effects of phenology after controlling for the other effects of polyploidy. By collecting data not only on flowering phenology but also on a range of other flower head characteristics, we were able to identify the contributions of the different traits to the fitness of the cytotypes. By linking the information on all the traits to seed production within each cytotype separately, we also aimed to assess the intensity of selection on the studied traits in each cytotype.

Specifically, we wanted to answer the following questions: (1) What is the effect of ploidy level, phenology and other flower head traits on the composition of insect communities in the flower heads? (2) What is the effect of ploidy level, phenology and other flower head traits on seed production? (3) Are the effects of ploidy level significant after accounting for phenology? and (4) Can the differences in the traits between the two cytotypes be explained by the intensity of selection on these traits?

To address these questions, we used a structural equation modelling approach to describe the relationships in the system using the combined data of the two cytotypes. Based on previous knowledge, we hypothesized that flowering phenology will be the most important factor driving seed production in the studied system. We also hypothesized that the differences in seed production between cytotypes could also be explained by flower head size and their position above ground and by the differential associations of the cytotypes with herbivores. In addition, we hypothesized that the effects of plant characteristics on seed production are mediated not only by herbivores but also by pollinators and by some other unstudied plant characteristics. Finally, we hypothesized that the different cytotypes experience different selection pressures, leading to trait differentiation between cytotypes. By using structural equation modelling on observational data, this study allowed us to investigate relationships between all the studied variables. Manipulative experiments, will, however, be needed in the future to confirm causality of these relationships.

## Methods

### Study system

*Centaurea phrygia* (sub-family Carduoideae, Asteraceae) is a perennial polycarpic plant species. In natural populations, it occurs in two main cytotypes, diploid (2*n* = 2*x* = 22 chromozomes) and tetraploid (2*n* = 4*x* = 44 chromozomes). The genetic diversity based on allozyme analyses and the morphological and genome size data suggest that the tetraploids are of autopolyploid origin (Koutecký *et al. *2012). The species occurs in Northern, Central and Eastern Europe. Diploids occur in Central and Northern European geographic range of the species except for the majority of the Western Carpathians. In contrast, the tetraploids are confined to a much smaller area in the Western Carpathians and adjacent areas. The distribution of both cytotypes is thus largely parapatric, with diploids and tetraploids co-occurring only in a limited contact zone in the intra-montane basins in the Western Carpathians (Koutecký *et al. *2012).

Both cytotypes are mainly outcrossing with very low seed production after selfing (Koutecký *et al.*, 2012). The peak flowering time of the tetraploids occurs in mid-June, while it occurs in mid-July for the diploids. In spite of this, the flowering period still largely overlaps between the cytotypes, providing extensive opportunities for between cytotype crossing (Münzbergová *et al. *2015). Common garden crossing experiments between the two cytotypes, however, suggested that the cytotypes are strongly reproductively isolated. Seed production after between-cytotype crosses was very low and with only very few triploid hybrids (Koutecký *et al. *2012).

*Centaurea phrygia* is, similar to other species from the subfamily Carduoideae, known to host a diverse insect community in the flower heads (e.g. Münzbergová et al., 2015). The seed herbivores in the flower heads of species of the subfamily Carduoideae usually belong to four main groups: weevils (Coleoptera, Curculionidae), fruit flies (Diptera: Tephritidae), tortrix moths (Lepidoptera: Tortricidae) and gall midges (Diptera: Cecidomyiidae) (Skuhrovec *et al. *2008; Koprdova *et al. *2015). The majority of these species are oligophagous, although some are polyphagous (e.g. Zwölfer *et al. *1971; Merz 1994; Skuhravá 1994). Some fruit flies occurring in the flower heads of some species of the subfamily are strict specialists (monophagous), e.g. *Terellia longicauda* on *Cirsium eriophorum* or *Urophora cardui* on *Cirsium arvense* (Merz 1994). No specialist is, however, known for *Centaurea phrygia.*

### Study populations

Information on the time of origin of the tetraploids and on the nature of the contact zone (primary or secondary) is important for the proper description of any diploid–polyploid system. However, no such information is available for this system. The range of distribution of the tetraploids is, however, much smaller than that of the diploids suggesting relatively recent origin. The two cytotypes have been treated as two separate species (diploid *C. phrygia* and tetraploid *C. erdneri*, Koutecký *et al. *2012). However, here we refer to the two entities as two cytotypes as in our previous study (Münzbergová *et al. *2015).

For the purpose of this study, we used three populations of each cytotype located in or very near the Czech Republic, Europe (one population is close to the Czech border in Poland) [**see Supporting Information—Table S1**]. These populations correspond to the populations previously examined in the study of Münzbergová *et al. *(2015). The populations of the two cytotypes were clearly separated from one another, with diploid populations coming from the central part of the country and tetraploid populations from the eastern part (for more details on vegetation and map see Münzbergová *et al. *2015). The information on the ploidy level of each natural population is provided in Koutecký *et al. *(2012), while the ploidy level of all the plants used in this study was confirmed by flow cytometry (see details below).

### Experimental design

Plants from seeds collected from the above-mentioned populations were grown at the Institute of Botany, Academy of Sciences in Průhonice, Czech Republic since May 2011, following plant cultivation and basic experimental set-up procedures described in Münzbergová *et al. *(2015). The plants used in this study are identical to those used in Münzbergová *et al. *(2015). This experiment was performed in 2014, i.e. 3 years after establishment of the plants. Thanks to this, maternal effects have been removed at least to some extent. In total, we had 20 plants from each population (60 plants of each cytotype) and grew each plant in a circular pot (19 cm in diameter, 20 cm deep) in an outdoor experimental garden. The pots were filled with a 1:1 mixture of common garden soil and sand. The plants were watered daily. All the pots were placed close to one another and the pots containing different cytotypes were completely intermixed. Before flowering, we assessed the ploidy level of all the plants using flow cytometry following the protocol described by Koutecký *et al. *(2012). The ploidy levels were consistent with the expected ploidy levels for the individuals and thus the flow cytometric analyses are not described further.

In the flowering season of 2014, we recorded flower head initiation and maturation for each flower head produced (678 flower heads in total). Flower head initiation was defined as the day at which the flower bud reached 2.5 mm in diameter. We assumed that flower buds at this size can be attacked by the first-arriving herbivores (Merz 1994; Volovnik 2016). At the time of flower head initiation, we recorded the number of simultaneously flowering flower heads within the same plant for each target flower head. Due to low values and low variation in the number of simultaneously flowering flower heads among individual plants as well as cytotypes (2.9 on an average), this parameter was excluded from the final analyses and is not mentioned further. The flower heads were monitored regularly, and when all the florets within a flower head had withered, that date was registered as the day of flower head maturation. The day of flower head maturation was the last possible occasion at which the insects could enter the flower heads (Merz 1994; Volovnik 2016). On the day of maturation, we recorded the flower head height above the ground and flower head height and width. Because these two measures were significantly correlated (*R*^2 ^=^ ^0.15, *P* < 0.001) and the correlation was not affected by ploidy level (*P* = 0.78), we only use flower head width in the subsequent analyses. Flower head width was chosen because it is a good proxy for number of ovules and it had better explanatory power in all preliminary analyses.

After recording the above-mentioned traits, each flower head was collected and placed in a rearing container. Each individual flower head was placed in a separate rearing container to collect herbivore interactions at the flower head level. The containers were inspected weekly and the emerging insects were removed. By removing the insects, we aimed to reduce the interactions between the different insects in the containers. While the removal interval could not fully eliminate these interactions, most of the insects that emerged were found dead in the container, suggesting that the conditions in the containers were suitable for their emergence but were not suitable for their subsequent survival. As a result, any insect–insect and insect–plant interactions in the containers were unlikely.

All the plants in the garden were inspected at 2 to 5-day intervals (with a 10-day interval in a few cases at the end of the flowering season) between the beginning of July and mid-September, i.e. over the period during which the plants flowered. The difference between the date of flower head maturation and the date of flower head initiation was used to calculate flower head longevity. This value represented the time over which the flower head could be colonized by herbivores.

After the emergence of all insects, each flower head was dissected and the number of developed undamaged seeds and damaged seeds were counted. In some cases, counting the number of damaged seeds was not possible due to their total destruction. In these cases, the number of damaged seeds was estimated based on the number of undamaged seeds and the receptacle size (correlation between receptacle size and seed production, *R*^2 ^=^ ^0.78, *P* < 0.001). These data were then used to estimate the proportion of damaged seeds. All the insects were separated into species, counted and identified with the help of experts for each of the insect taxa (see Acknowledgements).

### Data analyses

To assess the differences in flowering phenology between the cytotypes, we calculated the index of flowering overlap between them as suggested by Husband and Schemske (2000) using flower head initiation. The calculation was also made for flower head maturation, but since the patterns were similar to flower head initiation only index of overlap for flower head initiation is presented. We also used only flower head initiation as a measure of flowering phenology in the subsequent tests and SEM models described below.

We used analyses of variance and tested the effects of ploidy level, population nested within ploidy level and mother plant nested within population on the time of flower head initiation and flower head longevity. Similarly, we tested the effects of ploidy level, population and mother plant on the height of the flower head above the ground and the width of the mature flower head. We assumed a Gaussian distribution for all the dependent variables. In addition, we tested the effects of ploidy level, population nested within ploidy level and mother plant nested within population on the number of individuals and of species of all insect and of seed herbivores in particular, the number of developed undamaged seeds and the proportion of damaged seeds. In these cases, we assumed a Poisson distribution of the dependent variable, with the exception of the proportion of damaged seeds. The proportion of damaged seeds was evaluated using a binomial distribution after coding the number of damaged and undamaged seeds and linking these two using the cbind function in R. All the analyses were conducted using R 3.2.1 (R Development Core Team 2011).

**Figure 1:**
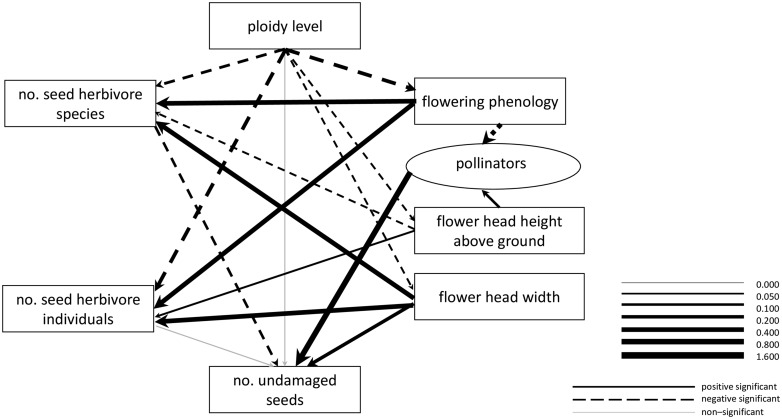
Path diagram describing the different factors affecting number of undamaged seeds in diploid-tetraploid *Centaurea phrygia*. Width of the line determines regression weights. The significance of each regression weight was judged based on overlap of 95 % credibility interval of the regression weight with 0. *N* = 678.

***Structural equation models*** **.** We used structural equation modelling (SEM, Scheiner and Callahan 1999; Scheiner *et al. *2000; Pugesek *et al. *2003), implemented in the software AMOS 5.0 (Small Waters Corporation, Chicago, IL, USA) to assess the relationship between flowering phenology (flower head initiation) and cytotype and the number of developed undamaged seeds. The full version of the SEM diagram including all the links that were originally explored is shown in [**See Supporting Information—Table S1**]. Due to the high number of links in the model, we dropped some of the links that were not significant. A simplified version of the model, in which we were able to properly estimate all the links and which is presented in the results, is shown in Fig. 1.

All the data entering the SEM models presented in this study are only observational. They thus allow assessing the likely relationships in the system but do not allow to explicitly test them. As a result of this, all the relationships detected in this study should be interpreted as likely links between the variables. Their causal relationships, however, have to be tested in future manipulative experiments.

Based on our previous knowledge of the system, we proposed that the association between ploidy level and the number of developed undamaged seeds is mediated by a wide range of factors including flowering phenology, flower head width and flower head height above the ground, the number of species and individuals of seed herbivores and other unstudied factors. While we also explored the possibility that the association between ploidy level and seed production is mediated by flower head longevity [**see Supporting Information—Table S1**], this was not confirmed, and flower head longevity was not included in the final model (Fig. 1). In addition, we also explored the possibility that ploidy level could be linked to the number of undamaged seeds via a latent variable (see below) representing the effect of pollinators [**see Supporting Information—Table S1**]. As this relationship was not confirmed, it was dropped from the final model (Fig. 1). Ploidy level, however, may be linked to the latent variable of pollinators via phenology and flower head height above the ground (see Fig. 1).

We also expected that ploidy level is related to the number of seed herbivore species and individuals. This relationship is likely mediated by phenology, flower head width and flower head height above the ground. We also included a direct link between ploidy level and the number of seed herbivore species and individuals into the model. This link represents differences between cytotypes in factors not evaluated in this study such as differences in the content of secondary metabolites (Münzbergová *et al. *2015).

Flowering phenology was expected to be linked to the number of developed undamaged seeds by its association to the number of species and individuals of seed herbivores and by interactions with pollinators. As we did not have any data on pollinators from the system, pollinators were treated as a latent variable in the model (Fig. 1). We also originally expected that flowering phenology will covary with flower head width and flower head height above the ground [**see Supporting Information—Table S1**], but this was not confirmed. Flowering phenology, flower head width and flower head height above the ground were thus independent of each other in the final model (Fig. 1).

The relationships among flower head width, flower head height above the ground and the number of developed undamaged seeds were expected to be mediated by the number of seed herbivore species and seed herbivore individuals. Flower head width was also linked directly to seed production. This path was included because flower head width is expected to be a proxy of the number of ovules, and consequently, seeds. Both flower head height above the ground and flower head width were linked to the latent variable of pollinators [**see Supporting Information—Table S1**]. As the relationship between flower head width and pollinators was not significant, it was dropped from the final model (Fig. 1).

All the data were mean-standardized before being input into the SEM. Ploidy level was coded as 0 for diploids and 1 for tetraploids. As a result, the slopes of the relationships between each pair of parameters in the path diagram indicated the percent of change in the target parameter, given a 1 % change in the affecting parameter. The issue of data standardization for the purpose of SEM has been extensively discussed in the literature. Grace and Bollen (2005) demonstrated that the most common approach is to use standard deviation-standardized data and suggested that unstandardized data should also be shown. Here, we decided to use mean-standardized data because such data allow for the interpretation of the slopes as a proportional change and consider the existing differences in variation of the different variables. Because the calculations were based on mean-standardized values, estimates of the slopes can have absolute values above 1. To express the overall effects of pathways, we combined the values for all the links within a pathway (Fig. 1).

In addition to comparing the two ploidy levels within one SEM model, we also performed a multigroup comparison as suggested by Grace (2003). To do this, we removed ploidy level from the SEM model and performed the analyses for each cytotype separately. These analyses provided information on the intensity of selection pressure for each cytotype.

For relationships with phenology, we examined models with only linear terms and models with quadratic terms. As the quadratic terms did not improve model fit, they are not discussed further. Because some data were not normally distributed, the significance of each link in the SEM model was assessed using Bayesian estimates (Lee 2007). The credibility intervals were then displayed. An overlap of the 95 % credibility interval with 0 was used to judge the significance of the link. A pathway was considered significant if all the links within that pathway were significant. For the multigroup comparison, we considered a link to be significantly different between the two cytotypes if the 95 % credibility intervals of the links did not overlap.

***Multivariate analyses*** **.** We used redundancy analyses (RDA), a form of linear multivariate analysis (Lepš and Šmilauer 2003) implemented in Canoco 4.0 (ter Braak and Šmilauer 1998), to study the determinants of the composition of insect communities in the flower heads. For all analyses, we excluded all species with less than three occurrences in the dataset. As some samples did not contain any insect species, we introduced a fictive species with one occurrence to all the samples. In this way, the empty samples were retained in the analyses but the fictive species did not have any effect on the results as long as we used linear analyses and did not standardize the data (ter Braak and Šmilauer 1998). First, we tested the separate effects of ploidy level, phenology, flower head size and flower head height above the ground on species composition. Afterwards, we used the significant variables as covariates and tested for the effects of ploidy level and phenology after accounting for flower head size and flower head height (in case they had significant separate effects) and the non-tested variable out of ploidy level and phenology (in case it had a significant separate effect).

## Results

### Flowering phenology and morphology

**Figure 2:**
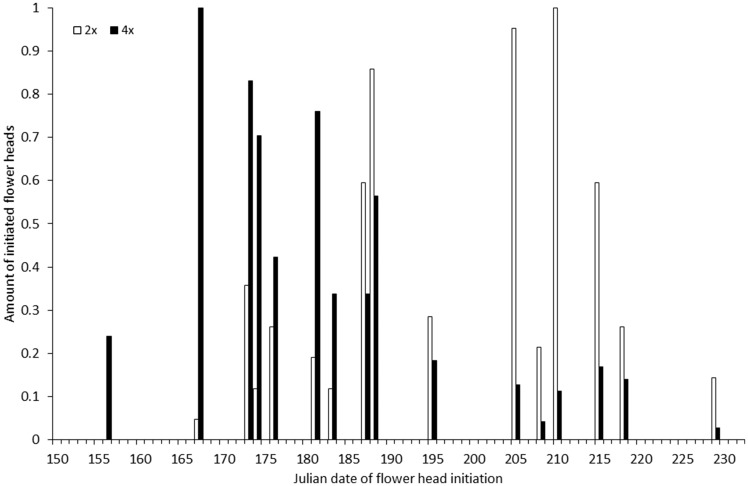
Flower head initiation in diploid and tetraploid individuals of *Centaurea phrygia* sampled in this study. The amount of flower heads is the number of flower heads initiated in the given day divided by the maximum number of initiated flower heads per day for the given cytotype.

The index of flowering overlap was 44.5 % (Fig. 2). Flower head initiation was significantly affected by ploidy level, population and mother plant, with population explaining much less variation (4.6 %) in flowering phenology than ploidy level (26.7 %) and mother plant (28.4 %, Table 1). Tetraploids were budding 18.8 days earlier than diploids (Fig. 2). In contrast, flower head longevity was independent of ploidy level. It was, however, affected by population and mother plant (Table 1).

**Table 1.** The effect of ploidy level, population nested within ploidy level and mother plant nested within population on flowering phenology, flower head longevity, flower head height above ground and flower head width tested using ANOVA. df error = 608. Significant values (*P* ≤ 0.05) are in bold.

		Flowering phenology			Longevity			Flower head above ground			Flower head width	
	Df	*F*	*P*	*F*		*P*	*F*		*P*	*F*		*P*
Ploidy level	1	**403.1**	**<0.001**	0.25		0.617	**54.3**		**<0.001**	**110.4**		**<0.001**
Population	4	**17.5**	**<0.001**	**6.65**		**<0.001**	**6.9**		**<0.001**	**2.9**		**0.02**
Plant	64	**6.7**	**<0.001**	**3.21**		**<0.001**	**7.1**		**<0.001**	**2.2**		**<0.001**

The flower heads of diploids occurred significantly higher above the ground than those of tetraploids (mean ± SE: 46.9 ± 0.99 cm, *N* = 223 and 38.9 ± 0.91 cm, *N* = 403, respectively; Table 1), but the difference was quite small (4.8 % variance explained). The flower heads of diploids were also significantly wider than the flower heads of tetraploids (43.0 ± 0.92 mm, *N* = 223 and 35.9 ± 0.54 mm, *N* = 403, respectively, 17 % variance explained, Table 1).

### Insect community diversity and composition

In total, 33 insect species belonging to 24 families were found in the flower heads [**see Supporting Information—Table S1**]. The species found in the flower heads included seed herbivores (6 species), carnivorous predators (6 species), parasitoids (12 species) and other species (9 species). The group marked as ‘other’ included diverse group of insects. Many of these insects are feeding on fungi (Coleoptera: Latridiidae, Throscidae) or pollen (Coleoptera: Nitidulidae, Leschen *et al. *2010). In addition, some species are phloem-sap feeding species (Sternorrhyncha: Aphididae) and a number of these species are most likely phytosaprophagous (Diptera: Chloropidae, Nartshuk, 2010; Š. Kubík, pers. comm.). The group ‘other’ also included species whose presence in the flower heads was likely accidental since these species develop on other plant species (e.g. Coleoptera: Curculionidae: *Ischnopterapion* and *Sitona* species, Koch 1992) or are most probably pollinators (e.g. Megachilidae).

The number of species and of individuals of all insects and of seed herbivores were significantly affected by ploidy level (Table 2). All variables except for the number of all insect species were also significantly affected by population and mother plant (Table 2). The number of all insect species was 1.5 times higher and number of all insect individuals was 2.1 times higher in flower heads of diploids than in tetraploids. The number of seed herbivore species was 2.3 times higher and the number of individuals of seed herbivores was 2.7 times higher in flower heads of diploids than in tetraploids.

**Table 2.** The effect of ploidy level, population nested in ploidy level and mother plant nested in population on number of insect individuals and species, number of seed herbivore individuals and species, number of undamaged seeds and proportion of damaged seeds in flower heads of *Centaurea phrygia*. The effects were tested using generalized linear model assuming Poisson distribution of the dependent variables (binomial distribution for proportion of damaged seeds). df error = 608. Significant values (*P* ≤ 0.05) are in bold. Dev. indicates deviance explained by the model.

		No. ins. species	No. ins. individuals	No. seed herb. species	No. seed herb. individuals	No. undamaged seeds	Prop. damaged seeds
Ploidy level	Dev.	**12.91**	**72.8**	**30.67**	**92.3**	**83.58**	**1.38**
df = 1	*P*	**<0.001**	**<0.001**	**<0.001**	**<0.001**	**<0.001**	**0.003**
	Higher in	**2×**	**2×**	**2×**	**2×**	**4×**	**2×**
Population	Dev.	4.24	**35.05**	**13.53**	**33.66**	**133.67**	0.58
df = 4	*P*	0.374	**<0.001**	**<0.001**	**<0.001**	**<0.001**	0.434
Plant	Dev.	5.33	**32.4**	**14.12**	**28.42**	**25.12**	0.25
df = 64	*P*	0.439	**<0.001**	**<0.001**	**<0.001**	**<0.001**	0.48

Insect community composition in the flower heads was significantly affected by phenology (*P* = 0.002, 2.1 % variance explained), ploidy level (*P* = 0.002, 2.9 % variance explained), flower head width (*P* = 0.002, 3.1 % variance explained) and height above the ground (*P* = 0.012, 0.5 % variance explained). The effect of ploidy level remained significant even after accounting for the effects of phenology, flower head width and flower head height above the ground (*P* = 0.002, 1.1 % variance explained). Seed herbivores *Chaetostomella cylindrica* and *Urophora* (s.str.) *quadrifasciata* and non-herbivorous *Orius niger* were more common in diploids, while seed herbivore *Acanthiophilus helianthi* and non-herbivorous species *Eurytoma* sp. 2, *Pteromalus* sp. 2, *Meligethes* sp. and Chrysopidae (Neuroptera) were more common in tetraploids. Similarly, the effect of phenology remained significant after accounting for ploidy level, flower head width and flower head height above the ground (*P* = 0.002, 1.0 % variance explained). Non-herbivorous species *Orius niger*, *Meligethes* sp. and Chrysopidae (Neuroptera) were more common in the early flowering flower heads, while seed herbivores *Acanthiophilus helianthi*, *Chaetostomella cylindrical* and *Urophora* (s.str.) *quadrifasciata* and non-herbivorous *Eurytoma* sp. 2 and *Pteromalus* sp. 2 were more common in the late flowering flower heads.

### Seed production and seed damage

**Figure 3:**
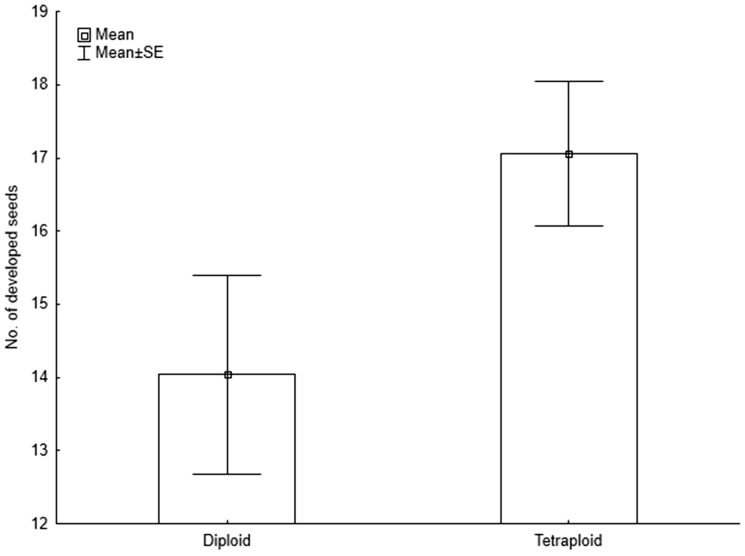
The effect of ploidy level on production of developed undamaged seeds. The difference in seed production between the two cytotypes is significant (*P* < 0.001) as shown in Table 2.

The generalized linear model exploring the effect of ploidy level on the number of developed undamaged seeds demonstrated significantly higher seed number in tetraploids than in diploids (Table 2 and Fig. 3). In addition, diploid plants also suffered significantly higher seed damage (Table 2).

Within the structural equation model, all links were significant except for the link between the number of seed herbivore individuals and the number of undamaged seeds and between ploidy level and the number of undamaged seeds (Fig. 1). The association between ploidy level and the number of undamaged seeds was thus mediated by phenology, height of the flower head above the ground, flower head width, the number of seed herbivore species and the latent variable of pollinators. Specifically, the effect of phenology mediated by the latent variable of pollinators led to a lower seed production in later-flowering flower heads. Later flowering flower heads also hosted more seed herbivore species and individuals, with the number of herbivore species further reducing seed production. All this resulted in higher seed production in early flowering plants, i.e. tetraploids and lower seed production in later-flowering plants, i.e. diploids (Fig. 1 and Table 3).

**Table 3.** Summary of the relationship of ploidy level and number of undamaged seeds in the flower heads estimated using structural equation modelling. The values shown are regression weights and indicate percentage of change in number of undamaged seeds given 1 % change in the studied factor. The relationship between ploidy level and number of undamaged seeds may go via phenology, flower head width and height above ground, number of seed herbivore species and individuals and other unexplored factors. These pathways may be mediated by seed herbivores, pollinators (used as latent variable) or other undescribed factors (depicted as direct link to proportion of damaged seeds or number of undamaged seeds, see Fig. 1). Negative values indicate higher seed production in diploids, positive values indicate higher seed production in tetraploids. Sum gives the summary of the relationships of the given set of pathways. Sum sig includes only the significant pathways. Significance of the single links is shown in Fig. 1.

	Relationship mediated by			Sum of the relationships	
Pathway via	Seed herbivores	Pollinators	Other	Sum	Sum sig
Phenology	0.025	0.146		0.170	0.163
Flower head width	0.020		−0.122	−0.101	−0.108
Flower head position	0.000	−0.028		−0.028	−0.029
No. seed herbivore species			0.009	0.009	0.009
No. seed herbivore ind.			0.017	0.017	0
Other			0.027	0.027	0
Overall				0.0943	0.0360

The association between flower head height and seed production mediated by the latent variable of pollinators resulted in a higher seed production in flower heads that were higher above the ground. Flower heads that were higher above the ground also hosted a higher number of seed herbivore individuals but a lower number of seed herbivore species. As the number of herbivore individuals in fact did not have a significant effect on seed production in the final model, this resulted in a higher number of undamaged seeds in flower heads that were higher above the ground, i.e. in diploids (Fig. 1 and Table 3).

Wider flower heads hosted a higher number of seed herbivore species and individuals. However, wider flower heads still produced a higher number of undamaged seeds, likely due to a higher number of ovules. This pathway thus suggested, similar to the height above the ground, higher seed production in diploids (Fig. 1 and Table 3). In contrast, the direct links between ploidy level and herbivores indicated that number of seed herbivore individuals and species was lower in tetraploid flower heads. As lower number of seed predator species led to higher seed production, this suggested a higher number of undamaged seeds in this cytotype (Table 3 and Fig. 1).

While the positive association between flower head width and flower head height above the ground suggested greater seed production in diploids, the negative association between flowering phenology and the number of seed herbivore species and individuals and seed production suggested the opposite (Table 3). Overall, the sum of the single estimates suggested a higher number of undamaged seeds in tetraploids (Table 3).

### Selection on plant characteristics in each cytotype

**Figure 4:**
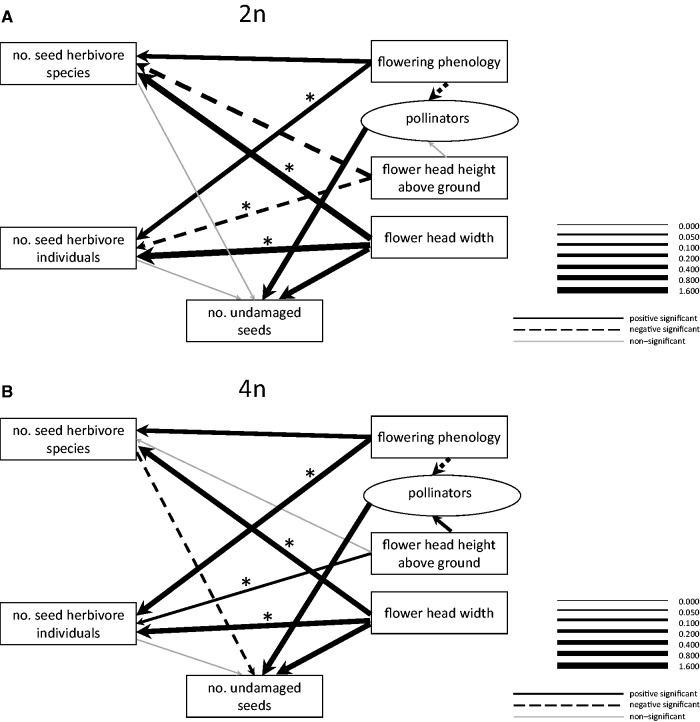
Path diagram describing the different factors affecting number of undamaged seeds in the system separately for (A) diploids and (B) tetraploids. Width of the line determines regression weights. Regression weight of the link between pollinators and number of developed undamaged seed was set to one in the model because pollinators represent a latent variable and the link thus could not be estimated from the data. The significance of each regression weight was judged based on overlap of 95 % credibility interval of the regression weight with 0. The significance of the difference between the regression weights for the two cytotypes was assessed based on overlap of their credibility intervals and is marked with asterisk (*) when credibility intervals do not overlap. Pollinators represent a latent variable in the model.

The strength of the associations among flowering phenology, the number of seed herbivore species and the latent variable of pollinators was comparable in both cytotypes. In contrast, the association between phenology and the number of seed herbivore individuals was much stronger in tetraploids than in diploids, with a much higher number of seed herbivore individuals in later-flowering flower heads (Fig. 4). As the number of seed herbivore individuals did not have significant effect on seed production in either cytotype, this in fact did not result in differential selection pressure on flowering phenology in the two cytotypes.

The strength of the associations among flower head height above the ground, the number of seed herbivore species and the latent variable of pollinators was not-significantly different between the two cytotypes (Fig. 4). Flower head height above ground is unlikely to be under selection due to its association with the number of seed predator species as one of the links to seed production was not significant in both cytotypes. In contrast, tetraploids may experience positive selection of flower head height aboveground as mediated by pollinators while diploids do not (Fig. 4). Flower head height above the ground was significantly positively associated with the number of seed herbivore individuals in tetraploids and was significantly negatively associated with the number of seed herbivore individuals in diploids (Fig. 4). As the number of seed predator individuals did not have any effect on seed production, this pathway did not result in selection on flower head height above the ground in either cytotype.

The number of seed herbivore species and individuals was significantly positively associated with flower head width in both cytotypes, but the increase in the number of seed herbivore species and individuals with increasing flower head width was significantly stronger in diploids. In spite of this, it can be expected that flower head width experienced negative selection in tetraploids and not in diploids as the only significant link between herbivores and seed production was for the number of herbivore species in tetraploids. The association between flower head width and the number of undamaged seeds did not significantly differ between the cytotypes (Fig. 4).

Overall, phenology was negatively associated with the number of undamaged seeds in both cytotypes, with a slightly stronger association in tetraploids when considering only the significant links (Table 4). In contrast, flower head width was positively associated with the number of undamaged seeds in both cytotypes. Flower head height above ground was not significantly linked to seed production in any of the cytotypes (Table 4).

**Table 4.** Results of structural equation models (regression weights) for each cytotype, i.e. diploid (2×) and tetraploid (4×), separately. The values shown are regression weights and indicate percentage of change in number of undamaged seeds given 1 % change in the studied factor. Number of developed undamaged seeds is determined by flowering phenology, flower head width and height above ground, number of seed herbivore species and individuals. These relationships may be mediated by seed herbivores, pollinators (used as latent variable) or other undescribed factors (depicted as direct link to proportion of damaged seeds or number of undamaged seeds, see Fig. 4). Sum gives summary of the relationships of the given set of pathways. Sum sig includes only the significant pathways. Significance of the single links is shown in Fig. 4.

		Effect mediated by			Sum of the effects	
Species characteristics		Seed herbivores	Pollinators	Other	Sum	Sum sig
Flowering phenology	2×	−0.085	−0.527		−0.612	−0.527
	4×	−0.089	−0.755		−0.844	−0.861
Flower head width	2×	−0.339		1.185	0.846	1.185
	4×	−0.126		1.545	1.419	1.401
Flower head height above g.	2×	0.052	0.183		0.235	0.000
	4×	0.001	0.347		0.348	0.000

## Discussion

The study demonstrated that the two ploidy levels of *C. phrygia* differed in terms of seed production. The relationship between cytotype and seed production occurred via flowering phenology, flower head width and position of the flower head above the ground, and the number of species and of individuals of seed herbivores. Some of these relationships were mediated not only by the performance of herbivores but also by that of pollinators. Because the link leading directly from ploidy level to the number of undamaged seeds was not significant, it can be expected that the studied factors are the main factors responsible for the relationship between cytotype and seed production with phenology having the strongest effect.

### Flowering phenology and morphology

Tetraploids in our study flowered significantly earlier than diploids. This is in line with our previous study (Münzbergová *et al. *2015), as well as Petit *et al. *(1997) and Nuismer and Cunningham (2005) observing other polyploid complexes, but contrasts with studies reporting earlier flowering in lower ploidy individuals (e.g. Jersáková *et al. *2010; Castro *et al. *2011). Earlier flowering in lower ploidy individuals may be explained by the slower development of polyploid plants (e.g. Otto and Whitton 2000; Oswald and Nuismer 2011) due to difficulties in the cell cycle and slower cell division (Comai 2005). In contrast, the earlier phenology of higher cytotypes could be caused by a larger cell size (Van Dijk and Van Delden 1990; Otto 2007) and the possible faster accumulation of biomass at later stages of plant development. Alternatively, the differences between cytotypes may be explained by their different evolutionary histories and may thus be unrelated to polyploidy (see discussion below).

In spite of the significant differentiation in phenology between the two cytotypes, there was still relatively high overlap between the flowering times of the two cytotypes. This high overlap indicates that flowering phenology is not an important pre-reproductive barrier between the cytotypes in this system (Husband and Sabara 2004). Flowering phenology was a strong predictor of seed production, with the number of developed undamaged seeds being lower in later-flowering plants. This is in line with the fact that phenology is a trait that is strongly related to fitness and is thus under strong, though sometime variable, selection pressure in a range of other systems (e.g. Nuismer and Ridenhour 2008; Ehrlén and Münzbergová 2009; Ehrlén *et al. *2015). This relationship was mediated by the higher number of seed herbivore species and individuals in the later-flowering flower heads, leading to an increased proportion of damaged seeds later in the field season. Additionally, the pathway via the latent variable of pollinators suggested that lower seed production occurred in later-flowering plants, likely due to a higher level of pollinator limitation later in the season.

The different pollinator and herbivore interactions along the flowering season observed here are in line with a recent review, which concluded that pollinators tend to be more active early in the field season and herbivores later in the field season (Elzinga *et al. *2007). The fact that the effects of phenology on the number of developed undamaged seeds are related to the life cycle and activity of insects, i.e. herbivores as well as pollinators, is in line with the fact that insects are known to be strongly affected by temperature (Schoonhoven *et al. *1998) due to their species-specific requirements for effective temperatures (Trudgill *et al. *2005; Martínková and Honěk 2008). In agreement with our study, lower seed production due to higher levels of seed damage later in the field season was also demonstrated in other systems (e.g. Ehrlén and Münzbergová 2009; Sletvold *et al. *2015), although the opposite pattern (Ehrlén *et al. *2015; Konig *et al. *2015) or no effect (e.g. Varga 2014; Konig *et al. *2015) have also been reported. Still, it is worth noticing that among these studies, only Konig *et al. *(2015) dealt with plants of different ploidy levels (tetraploid and octoploid *Cardamine pratensis*). A higher pollinator activity in the early or late part of the season has also been found (e.g. Hafdahl and Craig 2014; Bolmgren and Eriksson 2015; Kameyama and Kudo 2015). We are, however, not aware of any study directly combining the information on phenology and pollinator behaviour in different cytotypes in spite of a range of authors having studied pollinators and phenology within the same study (e.g. Jersáková *et al. *2010; Castro *et al. *2011; Borges *et al. *2012; Gross and Schiestl 2015). Selective pressures of pollinators and herbivores on flowering phenology along the flowering season are, thus, yet largely unexplored. Given the importance of phenology, they are in fact key aspects of the success of polyploid lineages.

The higher seed production of flower heads that were located higher above the ground may be related to the fact that flower heads that are higher are more visible to pollinators, and thus have a higher chance of being successfully pollinated (e.g. Gomez 2003; Červenková and Münzbergová 2014; Schlinkert *et al. *2015). At the same time, however, these flower heads may also be more visible to herbivores and be subjected to higher seed losses (e.g. Gomez 2003; Konig *et al.*, 2015; Schlinkert *et al. *2015). The resulting relationship between flower head height above the ground and seed production will thus be a result of the balance between pollinator limitation and herbivory in the system (Vanhoenacker *et al. *2013). In our dataset, the positive effects of higher levels of pollination prevailed over the negative effects of herbivores. In fact, herbivore had only weak on no effect on seed production. This suggests that pollinator interactions may be a more important factor determining final seed production in our system than seed herbivory. No direct data on pollinator communities and pollinator limitation and efficiency are, however, available for this system.

In contrast to previous studies suggesting that polyploids are larger than diploids due to their increased cell size scaling up to an increased size of plant organs (e.g. Lumaret 1988; Van Dijk and Van Delden 1990; Segraves and Thompson 1999; Ramsey and Schemske 2002), we demonstrated that diploid flower heads are larger than the flower heads of tetraploids (see also, e.g. Buggs and Pannell 2007; Černá and Münzbergová 2015; Münzbergová *et al. *2015 for a similar pattern). Wider flower heads were more attractive to herbivores but not to pollinators in our study. For herbivores, this is in line with a study by Rieder *et al. *(2001), suggesting that the larger flower heads of *Centaurea virgata* were more attractive to herbivores. In spite of these observations, wider flower heads produced more seeds. This is likely caused by a higher number of ovules in the wider flower heads that also provided disproportionally more seeds as a food source for herbivores. The absence of the relationship between flower head size and the latent variable pollinators is surprising given a range of previous studies demonstrating that plants with larger display are more attractive to pollinators (e.g. Grindeland *et al. *2005; Lobo *et al. *2016, but see Karron and Mitchell 2012). It thus seems that the pollinators in this system did not have any specific preferences for different flower size and the flower head height above the ground was a more importance driver of their preferences. Note, however, that we do not have any direct data on pollinators from this system. Such data are clearly necessary to understand the importance of pollinators in *C. phrygia*.

### Seed herbivores

The diversity and composition of the herbivore communities detected in this study were markedly different from those found in our previous study (Münzbergová *et al. *2015). In this study, the group of seed herbivores was represented mainly by tephritids and only partly by gall midges (Cecidomyiidae). The low number of gall midges and the absence of thrips (Thysanoptera: Thripidae) in the flower heads can be explained by a higher abundance of predators such as *Orius* species, which most likely consumed the gall midges. In addition, weevils (Curculionidae) and caterpillars (*Eucosma cana*, Lepidoptera: Tortricidae), which were responsible for most of the seed damage in the previous study, were not present in the samples analyzed here. The discrepancy in herbivore communities between the two studies may be a result of between-year variation in the synchronization between insect developmental times and flowering phenology.

Variations in composition and number of seed herbivores might lead to different behaviour of the different insect species possibly changing also their preferences. This may then strongly affect the fitness of the two cytotypes (Nuismer and Thompson 2001; Thompson *et al. *2004). The variation in the composition and diversity of herbivore communities may thus be responsible for the fact that tetraploids were distinctly more affected by herbivores than diploids in our previous study (Münzbergová *et al. *2015), while diploids suffered much higher seed damage in this study. In spite of this, diploids hosted more diverse and abundant seed herbivore communities in both studies (Münzbergová *et al. *2015 and results herein). The diversity and abundance of insects in the flower heads is expected to be affected by the content of various secondary metabolites acting as anti-herbivore agents. Previous studies, usually comparing diploids and synthetic polyploids (e.g. De Jesus-Gonzalez and Weathers 2003; Caruso *et al. *2011; Lavania *et al. *2012; Cohen *et al. *2013), demonstrated that higher ploidy levels are characterized by a higher content of secondary metabolites and thus better anti-herbivore defence, while opposite pattern was found only rarely (Repcak and Krausová 2009). In *C. phrygia*, six different metabolites occurring in higher concentrations in diploid flower heads have been detected, while only two metabolites were more abundant in the flower heads of tetraploids (Münzbergová *et al. *2015). This would suggest that diploids are better defended than polyploids and contrasts with most previous studies (references above) but also with the higher diversity and abundance of herbivores in the diploid flower heads observed here. Because the specific effects and targeted herbivore groups of each of the metabolites are unknown, it is, however, possible that the metabolites that are more abundant in tetraploids could be the two most important defensive chemicals acting against herbivores in *C. phrygia*.

Variation in the relationship between ploidy level and herbivore damage has also been seen in previous studies that have shown either greater seed damage in plants having a higher ploidy level (e.g. Münzbergová 2006a in *Aster amellus* and Arvanitis *et al. *2010 for gall midge damage in *Cardamine pratensis*), higher seed damage in plants having a lower ploidy level (e.g. Gross and Schiestl 2015 in *Gymnadenia conopsea*) or no effect of ploidy level (e.g. Boalt *et al. *2010 in *Cardamine pratensis*). This variation was also confirmed in several studies of *Heuchera grossulariifolia* (e.g. Nuismer and Thompson 2001; Thompson *et al. *2004) where different herbivores preferentially fed on different cytotypes, leading to greater seed damage in diploids after attack by one seed herbivore and greater seed damage in tetraploids after attack by another seed herbivore. In addition, Halverson *et al. *(2008) demonstrated that plant-herbivore associations in different cytotypes of *Solidago altissima* are not only affected by year and herbivore species, but they also differ between plant populations. This thus suggests that a change in herbivore community composition may have dramatic effects on the outcome of plant–herbivore interactions and consequently on plant fitness. Knowledge of the specific composition of herbivore communities is thus essential to properly understand the possible determinants of herbivore damage in each specific case and to properly interpret the results.

### Comparison of the cytotypes and selection on plant characteristics

Wider flower heads and a higher position of the flower heads above the ground suggested higher seed production in diploids, while phenology and the number of seed herbivores suggested the opposite. Taken together, there was significantly higher seed production in tetraploids compared to diploids. This is in agreement with several previous studies demonstrating higher seed production in plants of higher ploidy level (e.g. Münzbergová 2006a; Černá and Münzbergová 2013; Eliášová *et al. *2014; Gross and Schiestl 2015), but other studies showed an opposite effect (Burton and Husband 2000, 2001) or no difference (Münzbergová 2007; Castro *et al. *2011). The high variability in the outcomes of the previous studies can be explained by contrasting effects of ploidy level on seed production via different plant characteristics, as found in our study. Additionally, the effects of ploidy level on seed production may vary between years, as seed production depends on a wide range of factors including flowering phenology and the performance of pollinators and seed herbivores. Data from multiple years should thus be collected to detect general patterns in the effects of ploidy level on seed production.

The comparison of the SEM models that were created separately for each cytotype demonstrated potential selection for earlier flowering in both cytotypes, with selection being stronger in the tetraploids. This selection was mediated mainly by the latent variable of pollinators and in tetraploids also by seed herbivores. The effect of seed herbivores was, however, weaker than that of pollinators. Stronger selection in tetraploids is in line with the fact that tetraploids flower earlier than diploids. The only previous study explicitly looking at the intensity of selection pressures in different cytotypes also demonstrated selection for earlier flowering in tetraploids and that this selection was mediated by an herbivore (Nuismer and Ridenhour 2008). However, later flowering plants were selected for in diploids in their study and this effect was mediated by other unknown factors.

In both cytotypes in the present study, there was also strong selection for wider flower heads, with a stronger effect in tetraploids. Finally, there seems to be only little selection pressure incurred by seed herbivores, suggesting that the selection for better defence against seed herbivores might have been weak in the studied year. It was in fact significant only in tetraploids. The lack of congruence between observed species characteristics and the direction of selection may be explained by the fact that selection pressure is known to strongly vary between years in terms of both intensity and direction (e.g. Nuismer and Ridenhour 2008; Ehrlén and Münzbergová 2009; Konig *et al. *2015). Additionally, these traits may be affected by other unstudied selection pressures, or their values may be due to differential selection pressures of the past.

### Limitations of the study

The material used in this study came from populations of diploids and tetraploids with parapatric distributions. All the patterns observed in this study and interpreted as differences between the two cytotypes may thus not be a direct consequence of polyploidy but rather a consequence of differential evolutionary history. The impossibility to separate the effects of polyploidy from the effects of subsequent evolution of the polyploid taxa is in fact a problem in the majority of existing ecological studies on polyploids. The few existing exceptions used synthetic polyploids to enable such a separation and have indeed demonstrated that newly created synthetic polyploids differ from established polyploids in wide range of traits (Husband *et al. *2008; Maherali *et al. *2009; Oswald and Nuismer 2011). No synthetic polyploids are, however, available for our study system, and such a separation is thus not possible. The results of this study should therefore be interpreted with caution.

This study occurred in an experimental garden at the edge of the ranges of distribution of the studied species, where we created an artificial mixed ploidy population. Because the species are mostly parapatric and there are only very few mixed ploidy populations, this study does not fully represent the processes that occur under natural conditions. Instead, it simulates a theoretical case of a secondary contact zone of the species and describes the possible interactions between the two cytotypes in such a contact zone.

While this study demonstrated many significant determinants of seed production in our system, it did so only for a single year (2014). When comparing these results with the results from our previous study (Münzbergová *et al. *2015) in the same system that took place in 2011 and 2012, it is evident that although the general patterns hold across studies, the patterns in seed damage and herbivore communities were largely different between the two studies. This is in line with other studies, which showed that between-year variation may play an important role when assessing the direction of the complex relationships affecting plant fitness (Ehrlén and Münzbergová 2009; Munguia-Rosas *et al. *2011). Studies over multiple years are thus needed.

The main response variable used in our study was the number of developed undamaged seeds produced by a single flower head. While seed production is regularly considered to be a proxy of plant fitness in a range of other studies (e.g. Campbell and Powers 2015; Lehndal and Agren 2015), several studies have suggested that seed production may not be an optimal fitness measure, as fitness also depends on seed germination, seedling establishment, and the ability of plants to reach reproductive status (e.g. Münzbergová 2005; Münzbergová 2006b; Dostálek and Münzbergová 2013). In addition, because the plants were cultivated in an artificial mixed-ploidy setting in the garden, they may experience intercytotype crossing. The seed production of the plants could thus be affected by asymmetrical hybridization because of a lack of or biased pollinator preference, asymmetrical pollen competition within stigmas, or asymmetrical triploid abortion, although strong gametic isolation has been observed among the cytotypes of *C. phrygia* (Koutecký *et al. *2012).

It should also be noted that this study is based solely on observational data. All the relationships deduced and presented in the study thus provide insights into the possible relationships in the system, but do not imply causality. Manipulative experiments will thus be needed in the future to confirm that the relationships presented here are really causal.

## Conclusions

The study demonstrated that the relationship between ploidy level and seed production is mediated by a wide range of factors, including flowering phenology, flower head width and the flower head height above the ground, and association of the flower heads with herbivores and pollinators. The relationship of cytotype and seed production via the different pathways goes in opposing directions. Overall, the study demonstrated that tetraploids possess overall higher fitness as estimated by their seed production. Previous study on the same species carried out in different years, however, indicated higher seed production in diploids. This discrepancy is likely caused by the fact that final seed production is a result of many different interacting factors such as phenology, morphology, pollinator activity and insect damage often with contrasting effects and with interactions varying over time. The resulting difference in seed production between cytotypes can thus be a result of subtle changes in the importance of the different interacting factors. We thus suggest that knowledge of the structure of the specific processes affecting final seed production is more important for understanding the specific system and for designing models aimed to understand the dynamics of diploid–polyploid systems than the overall effects of cytotype on the fitness trait. The analyses in this study provided the conceptual framework describing the possible relationships in our system. Manipulative experiments are now needed to link these relationships to causal effects.

## Supplementary Material

Supplementary Data

## Supporting Information

The following additional information is available in the online version of this article —

**Table S1.** Positions of the source localities of the plant material and of the experimental garden.

**Table S2.** List of insect species found in the flower heads of diploid (2x) and tetraploid (4x) *Centaurea phrygia*. The values show number of individuals of each species per one flower head. The larger of the two values per species is in bold. – indicates that the species is not present. Column prev. indicates whether the given species was detected also

**Figure S1.** Diagram showing all the possible links tested within the SEM model in the initial tests. All the non-significant links and variables (shown in grey, except for the direct link between ploidy level and seed production) were dropped from the final model to ensure that all the links could be properly identified.
