# Supplementary material for: Contrasting effects of ploidy level on seed production in a diploid–tetraploid system
Source: AoB Plants. 2016 Dec 24;9(1):plw077. doi: 10.1093/aobpla/plw077 (PMC5499820; doi:10.1093/aobpla/plw077)

**SUPPORTING INFORMATION**

Table S1. Positions of the source localities of the plant material and of the experimental garden.

|  | Source populations | | | | | | Experimental garden |
| --- | --- | --- | --- | --- | --- | --- | --- |
| Ploidy | 2x | | | 4x | | |  |
| Elevation (m a.s.l.) | 430 | 290 | 310 | 490 | 500 | 690 | 319 |
| N | 50.9303 | 50.3391 | 50.3242 | 49.5454 | 49.5826 | 50.0175 | 49.9955 |
| E | 15.3684 | 17.1254 | 17.1994 | 18.6791 | 18.4803 | 17.3042 | 14.5666 |
| Country | PL | CZ | CZ | CZ | CZ | CZ | CZ |

Table S2. List of insect species found in the flower heads of diploid (2x) and tetraploid (4x) *Centaurea phrygia*. The values show number of individuals of each species per one flower head. The larger of the two values per species is in bold. – indicates that the species is not present. The column ‘prev.’ indicates whether the given species was also detected in our previous study in the system (Münzbergová et al., 2015).

| ORDER | FAMILY | SPECIES | 2x | 4x | prev. |
| --- | --- | --- | --- | --- | --- |
| SEED HERBIVORES | |  |  |  |  |
| Diptera | Tephritidae | *Acanthiophilus helianthi* (Rossi, 1790) | **0.209** | 0.185 | 1 |
| Diptera | Tephritidae | *Chaetostomella cylindrica* (Robineau-Desvoidy, 1830) | **0.558** | 0.146 | 1 |
| Diptera | Tephritidae | *Urophora* (s.str.) *quadrifasciata* (Meigen, 1826) | **0.213** | 0.038 | 1 |
| Diptera | Cecidomyiidae | *Dasineura* sp. | - | 0.002 | 1 |
| Lepidoptera | Tortricidae | *Eucosma cana* (Haworth, 1811) | - | 0.002 | 1 |
| Heteroptera | Miridae | *Lygus rugulipennis* Poppius 1911 | - | 0.005 | 0 |
| PARASITOIDS |  |  |  |  |  |
| Hymenoptera | Eurytomidae | *Eurytoma* sp. 2 | 0.008 | **0.012** | 1 |
| Hymenoptera | Eurytomidae | *Eurytoma* sp. 3 | 0.004 | - | 0 |
| Hymenoptera | Pteromalidae | *Pteromalus* sp. 2 | 0.004 | **0.012** | 1 |
| Hymenoptera | Eulophidae | *Tetrastichus* sp. 1 | - | 0.002 | 1 |
| Hymenoptera | Eulophidae | *Tetrastichus* sp. 2 | - | 0.022 | 0 |
| Hymenoptera | Ceraphronidae | gen. sp. | - | 0.007 | 0 |
| Hymenoptera | Scelionidae | gen. sp. | 0.004 | - | 0 |
| Hymenoptera | Eucoilidae | *Leptopilina* | 0.004 | - | 0 |
| Hymenoptera | Braconidae | gen. sp. 1 | 0.004 | - | 1 |
| Hymenoptera | Braconidae: Aphidiinae | gen. sp. | - | **0.005** | 0 |
| Hymenoptera | Ichneumonidae: Diplazontinae | gen. sp. 1 | - | **0.002** | 0 |
| Hymenoptera | Ichneumonidae: Diplazontinae | gen. sp. 2 | - | **0.002** | 0 |
| PREDATORS |  |  |  |  |  |
| Heteroptera | Anthocoridae | *Orius niger* (Wolff, 1811) | **0.076** | 0.031 | 1 |
| Heteroptera | Anthocoridae | *Orius* (*Heterorius*) sp. | 0.004 | - | 0 |
| Neuroptera | Chrysopidae | gen. sp. | 0.004 | 0.010 | 0 |
| Hymenoptera | Formicidae | *Lasius niger* (Linneaus, 1758) | - | 0.002 | 0 |
| Hymenoptera | Formicidae | *Myrmica rubra* (Linneaus, 1758) | 0.004 | 0.004 | 0 |
| Araneae | Araneidae | gen. sp. | 0.133 | 0.002 | 0 |
| VARIOUS |  |  |  |  |  |
| Coleoptera | Nitidulidae | *Meligethes* sp. | 0.044 | **0.141** | 1 |
| Coleoptera | Curculionidae | *Ischonopterapion virens* (Herbst, 1797) | - | 0.002 | 0 |
| Coleoptera | Curculionidae | *Sitona* *lineatus* (Linneaus, 1758) | - | 0.002 | 0 |
| Coleoptera | Latridiidae | *Corticaria*. sp. 3 | 0.012 | - | 0 |
| Coleoptera | Throscidae | gen. sp. | 0.004 | - | 0 |
| Diptera | Chloropidae | *Polyodaspis* *ruficornis* (Macquart, 1835) | 0.004 | 0.002 | 1 |
| Diptera | Anthomyiidae | gen. sp. | - | 0.002 | 0 |
| Hymenoptera | Megachilidae | *Pseudoanthidium* *lituratum* (Panzer, 1801) | - | 0.002 | 0 |
| Sternorhyncha | Aphididae | gen. sp. | 0.004 | **0.022** | 0 |

Figure S1. Diagram showing all the possible links tested within the SEM model in the initial tests. All the non-significant links and variables (shown in grey, except for the direct link between ploidy level and seed production) were dropped from the final model to ensure that all the links could be properly identified.


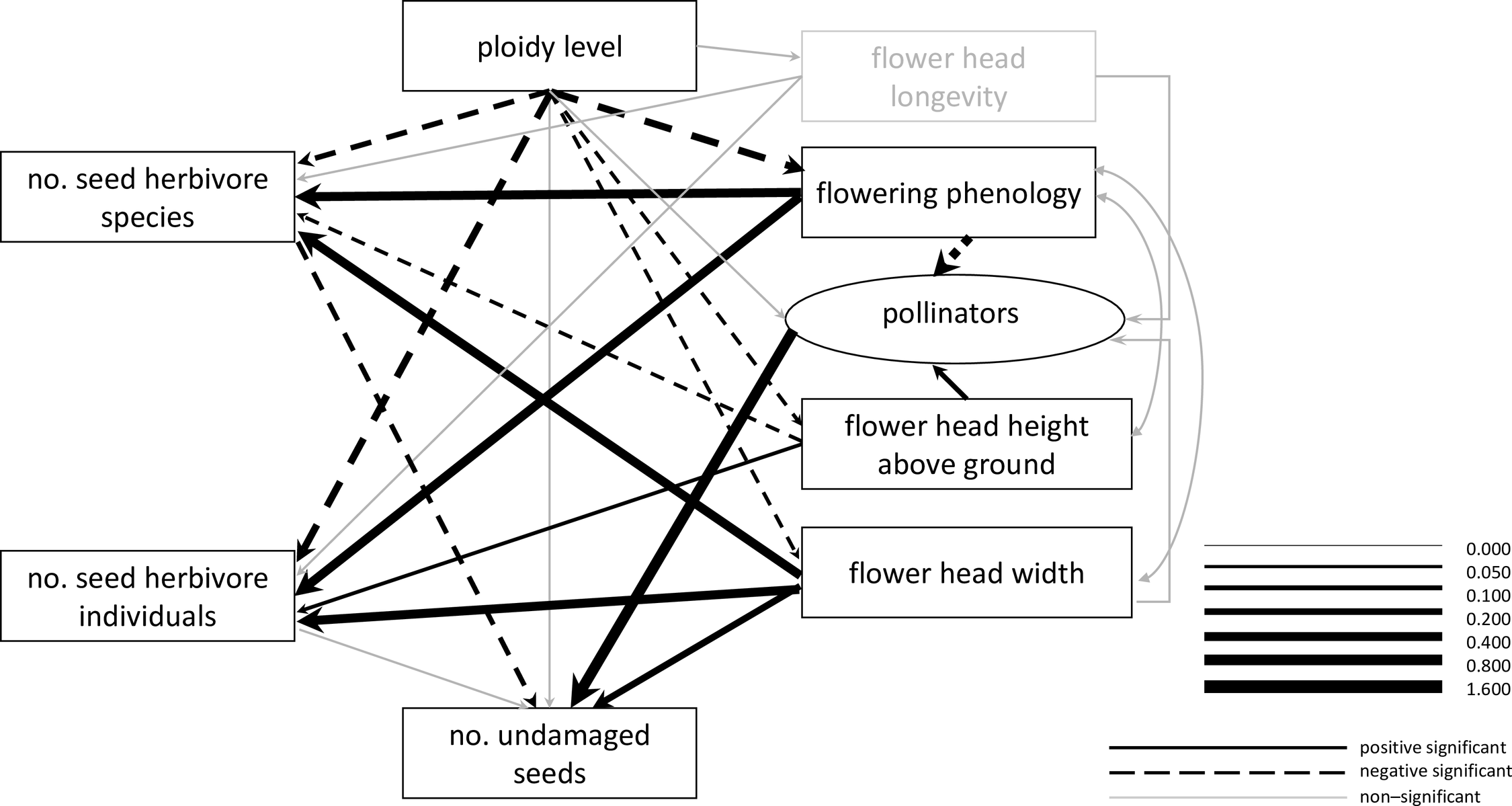

Supplement: Supplementary Data [file plw077_Supp.docx]
